# Defatted Kenaf (*Hibiscus cannabinus* L.) Seed Meal and Its Phenolic-Saponin-Rich Extract Protect Hypercholesterolemic Rats against Oxidative Stress and Systemic Inflammation via Transcriptional Modulation of Hepatic Antioxidant Genes

**DOI:** 10.1155/2018/6742571

**Published:** 2018-04-22

**Authors:** Kim Wei Chan, Maznah Ismail, Norhaizan Mohd Esa, Noorjahan Banu Mohamed Alitheen, Mustapha Umar Imam, Der Jiun Ooi, Nicholas M. H. Khong

**Affiliations:** ^1^Institute of Bioscience, Universiti Putra Malaysia, 43400 Serdang, Selangor, Malaysia; ^2^Department of Nutrition and Dietetics, Faculty of Medicine and Health Sciences, Universiti Putra Malaysia, 43400 Serdang, Selangor, Malaysia; ^3^Department of Cell and Molecular Biology, Faculty of Biotechnology and Biomolecular Sciences, Universiti Putra Malaysia, 43400 Serdang, Selangor, Malaysia; ^4^Department of Medical Biochemistry, Faculty of Basic Medical Sciences, College of Public Health, Usmanu Danfodio University, PMB 2346, Sokoto, Nigeria

## Abstract

The present study aimed to investigate the antioxidant and anti-inflammatory properties of defatted kenaf seed meal (DKSM) and its phenolic-saponin-rich extract (PSRE) in hypercholesterolemic rats. Hypercholesterolemia was induced using atherogenic diet feeding, and dietary interventions were conducted by incorporating DKSM (15% and 30%) or PSRE (at 2.3% and 4.6%, resp., equivalent to the total content of DKSM-phenolics and saponins in the DKSM groups) into the atherogenic diets. After ten weeks of intervention, serum total antioxidant capacities of hypercholesterolemic rats were significantly enhanced by DKSM and PSRE supplementation (*p* < 0.05). Similarly, DKSM and PSRE supplementation upregulated the hepatic mRNA expression of antioxidant genes (Nrf2, Sod1, Sod2, Gsr, and Gpx1) of hypercholesterolemic rats (*p* < 0.05), except for Gpx1 in the DKSM groups. The levels of circulating oxidized LDL and proinflammatory biomarkers were also markedly suppressed by DKSM and PSRE supplementation (*p* < 0.05). In aggregate, DKSM and PSRE attenuated the hypercholesterolemia-associated oxidative stress and systemic inflammation in rats, potentially by enhancement of hepatic endogenous antioxidant defense via activation of the Nrf2-ARE pathway, which may be contributed by the rich content of phenolics and saponins in DKSM and PSRE. Hence, DKSM and PSRE are prospective functional food ingredients for the potential mitigation of atherogenic risks in hypercholesterolemic individuals.

## 1. Introduction

Cardiovascular diseases (CVDs) remain as the leading cause of global mortality for the past 15 years. In 2015, CVDs had claimed 17.7 million lives, accounting for approximately 45% of all noncommunicable diseases deaths and 31% of all deaths globally [1]. Atherosclerosis is the core pathological element that underlies CVDs, contributing to over 80% of CVD-related fatalities worldwide [2]; while hypercholesterolemia is one of the most prominent risk factors for developing atherosclerosis [3]. Although hypercholesterolemia is related to excessively elevated levels of circulating total and non-high-density lipoprotein cholesterols in the blood, it is not solely a metabolic disorder of cholesterol homeostasis. Instead, hypercholesterolemia is indispensably associated with exacerbation of oxidative stress and inflammation, which culminates in the impairment of vascular reactivity and progression of atherogenesis [4]. The hypercholesterolemic environment activates major oxidant-producing enzymes including xanthine oxidase, NADPH oxidases (NOX), and myeloperoxidase, resulting in the excessive generation of reactive oxygen species (ROS) and oxidative stress consequently. Oxidative stress abrogates endothelial nitric oxide (NO) availability, uncouples endothelial nitric oxide synthase (eNOS), and enhances the oxidation of entrapped low-density lipoprotein (LDL) within the subendothelial space, thereby eliciting the vascular inflammation response by recruiting monocytes into the tunica intima. The unregulated uptake of oxidized LDL (oxLDL) by differentiated monocytes (macrophages) leads to the formation of foam cells, producing numerous proinflammatory and oxidative stress markers, cytokines, and growth factors, which further aggravate the atherogenic process [4–7].

Improvements in awareness of CVDs, their risk factors, and preventive behaviors have been evident around the world. The appeal in the relationship between diet and health coupled with consumer acceptance for the concept of functional foods, and better understanding of its determinants, has stimulated exponential growth of the global functional food market recently. According to the latest market report, the global cardiovascular health market was valued at USD 8.2 billion in 2016 [8]. Due to the strong correlation between hypercholesterolemia, oxidative stress, and inflammation in the pathogenesis of atherosclerosis, the search for cardioprotective functional food ingredients that possess strong antioxidant and anti-inflammatory properties in addition to cholesterol-lowering effects is receiving increasing attention from related authorities, researchers, manufacturers, and consumers [9–13].

Kenaf (*Hibiscus cannabinus* L.) is a commercial fibre crop, cultivated mainly for its stem and stalk for the production of biocomposites, paper, fibre boards and bioplastics, and biofuel. Kenaf seed is one of the major by-products from the kenaf plantation, and its seed oil has been extensively studied for its potential as functional edible oil [14–17]. Defatted kenaf seed meal (DKSM) is the secondary waste product yielded from the kenaf seed oil extraction process, which accounts for over 75% of its seed mass. Recently, DKSM has been increasingly proven and attested to be a novel functional edible flour with highly nutritive, antioxidative, antihypercholesterolemic, and anticancer properties. Furthermore, our findings also showed that phenolics and saponins are the two major bioactives in DKSM that correspond to the aforementioned nutraceutical properties [18–22]. Aside from the previous reports on antioxidant properties of DKSM and its bioactive-rich extract based on chemical assays, their antioxidant effects under physiological or pathological conditions have not been delved into. Furthermore, studies of anti-inflammatory properties of DKSM and PSRE in a hypercholesterolemic *in vivo* model have not been reported hitherto. Hence, the objectives of the present study were to investigate the antioxidant and anti-inflammatory properties of DKSM and PSRE supplementation via a hypercholesterolemic rat model. In addition, their modulatory effects on the hepatic mRNA level of antioxidant genes were also studied. PSRE was prepared and tested along with DKSM at the equivalent levels of total DKSM-phenolics and saponins in order to determine the possible contributing roles of both bioactives in the *in vivo* antioxidant and anti-inflammatory properties of DKSM. To date, this is the first study to report on the antioxidant and anti-inflammatory properties of DKSM and PSRE supplementation in a hypercholesterolemic rat model.

## 2. Materials and Methods

### 2.1. Materials

Ingredients of rat diets, that is, standard rat chow, cholesterol, cholic acid, palm oil, corn starch, full cream milk powder, and eggs, were purchased from Specialty Feeds (Glen Forrest, Australia), Amresco (Solon, OH, USA), Santa Cruz Biotechnology Inc. (Dallas, TX, USA), Yee Lee Edible Oil Sdn. Bhd. (Perak, Malaysia), Thye Huat Chan Sdn. Bhd. (Penang, Malaysia), Eaga Exports Pty Ltd. (South Perth, Australia), and Lay Hong Berhad (Klang, Selangor, Malaysia), respectively. Simvastatin was purchased from Pfizer (New York, NY, USA), while potassium persulfate, 6-hydroxy-2,5,7,8-tetramethylchroman-2-carboxylic acid (Trolox), and 2,2′-azino-bis(3-ethylbenzthiazoline-6-sulphonic acid) (ABTS) were purchased from Sigma-Aldrich Co. (St. Louis, MO, USA). All solvents of analytical grade were purchased from Merck (Darmstadt, Germany). Fixative solution (RCL2®) was purchased from Alphelys (Plaisir, France). Rat oxidized low-density lipoprotein (oxLDL) and interleukin 6 (IL-6) ELISA kits were purchased from Cusabio (Wuhan, Hubei, China), while rat tumour necrosis factor-alpha (TNF-*α*) and C-reactive protein (CRP) ELISA kits were purchased from EMD Millipore, Merck (Darmstadt, Germany). GenomeLab™ GeXP Start Kit and RNA isolation kit (GF-TR-100 RNA Isolation Kit) were purchased from Beckman Coulter Inc. (Brea, CA, USA) and Vivantis (Selangor, Malaysia), respectively. Magnesium chloride (MgCl_2_) and DNA Taq polymerase were purchased from Thermo Fisher Scientific (Pittsburgh, PA, USA).

### 2.2. Preparation of DKSM and PSRE

Kenaf seeds (variety V 36) were obtained from the Malaysian Kenaf and Tobacco Board in Pasir Putih, Kelantan, Malaysia, and DKSM was produced following the defatting procedures of our previous study [19]. Briefly, ground kenaf seeds were homogenized at 9500 rpm (Ultra-turrax T25 basic, IKA®-WERKE GmbH & Co. KG, Staufen, Germany) with n-hexane at the ratio of 1 : 2 (*w* : *v*) for 15 min. Then, the mixture was filtered through Whatman number 2 filter paper. The residue (DKSM) was reextracted twice accordingly and dried in an oven at 50°C for 3 h to remove residual solvent. Finally, DKSM was passed through a 30-mesh sieve and kept in −20°C for further use. Proximate analysis showed that DKSM contained 57.09% carbohydrate, 26.19% protein, 9.34% moisture, 6.65% ash, and 0.73% fat [19]. Besides, DKSM also contained 16.95% crude fibre.

Phenolic-saponin-rich extract (PSRE) containing the total phenolics and saponins of DKSM was prepared according to the extraction procedures in our previous work [18]. In brief, DKSM was refluxed in 50% aqueous ethanol for 3 h in the ratio of 1 : 15 (*w* : *v*). Then, the mixture was filtered through Whatman filter paper number 2. Finally, solvents in the filtrate were evaporated under reduced pressure (Rotavapor R210, Buchi, Flawil, Switzerland) followed by lyophilization (VirTis BenchTop K Freeze Dryer, SP Industries, Warminster, PA, USA) to obtain PSRE. In order to estimate the recoveries of DKSM-phenolics and saponins in PSRE, DKSM residue obtained from the aforementioned procedure was extracted with methanol under sonication for 1 h. Then, the mixture was centrifuged at 7500 rpm for 10 min at 25°C. Subsequently, the supernatant was subjected to determination of total phenolic and saponin contents, respectively, by Folin–Ciocalteu reagent and vanillin-sulphuric acid assays [18, 23, 24]. The recoveries of phenolics and saponins in PSRE from DKSM were estimated at 97.2 ± 0.1% and 92.5 ± 1.8%, respectively.

Characterization of targeted bioactives in PSRE, that is, phenolics and saponins, was reported in our previous work [18], and the same batch of DKSM and PSRE was used in the present study. From our study [18], total phenolic content of PSRE was estimated at 34.44 mg/g sample, with *p*-coumaric acid (27.72 mg/g sample), caffeic acid (5.75 mg/g sample), (+)-catechin (0.86 mg/g sample), and gallic acid (0.11 mg/g sample) detected as the major phenolics present. Besides, PSRE was found to contain total and steroidal saponins of 128.66 and 0.83 mg diosgenin equivalents/g sample, respectively. Correspondingly, DKSM contains about 5.29 mg/g sample of total phenolics, which was composed of 4.26 mg of *p*-coumaric acid, 0.88 mg of caffeic acid, 0.13 mg of (+)-catechin, and 0.02 mg of gallic acid. Total saponin and steroidal saponin contents of DKSM were estimated at 19.76 and 0.13 mg diosgenin equivalents/g sample, respectively.

### 2.3. Animal Study

Approval for the animal study was granted by the Institutional Animal Care and Use Committee (IACUC) of Universiti Putra Malaysia (Animal Ethics Approval Number: UPM/IACUC/AUP-R065/2013). The study was conducted in accordance with the guidelines for the use of animals. Forty-two male Sprague-Dawley rats (6 weeks old, 130–150 g) were housed in individual plastic cages under the controlled condition of 12/12 h light/dark cycle, at 25 to 30°C. During the acclimatization period (1 week), all rats were fed with standard rat chow (*ad libitum*) and given free access to water. After that, the rats were randomly assigned into 7 different groups, each consisting of 6 rats, that is, NC: rats were fed with standard rat chow; AD: rats were fed with an atherogenic diet containing 20% palm oil, 2% cholesterol, and 0.4% cholic acid; DKSM-Low and DKSM-High: rats were fed with a similar diet to the AD group except for the replacement of rat chow with DKSM (15% and 30% of total diet, resp.); PSRE-Low and PSRE-High: rats were fed with a similar diet to the AD group except for the replacement of rat chow with PSRE respectively at the level of 2.3% and 4.6%, of the total diet (based on 15.36% extraction yield from DKSM, which are corresponded to the equivalent levels of total DKSM-phenolics and saponins in the DKSM groups); and Statin: rats were fed with an atherogenic diet and administrated with simvastatin (10 mg/kg body weight/day) through oral gavage. All diet compositions and caloric values are depicted in Table 1. As shown in Table 1, replacement of DKSM and PSRE with rat chow did not significantly alter the energy distribution of atherogenic diets. Food was given based on daily isocaloric value of 30 kcal/100 g body weight for 10 weeks, and prefiltered tap water was supplied in water-dispensing bottles *ad libitum*. After 10 weeks of dietary intervention, all rats were euthanized (exsanguination by cardiac puncture under anesthesia by ketamine (100 mg/kg) and xylazine (10 mg/kg)) after an overnight fast. Fasting sera were obtained via centrifugation of collected bloods. Rats' livers were carefully excised, cleaned, and preserved in RCL2® solution at −80°C.

The effects of DKSM and PSRE supplementation on the cholesterol metabolism of experimental rats from the present study have been reported [22]. Biochemical analysis on rats' sera showed that atherogenic diet feeding had successfully induced hypercholesterolemia and liver steatosis in rats, as evidenced by significant elevations in hepatosomatic index and hepatic lipid content as well as levels of circulating total and LDL cholesterol, as compared to the NC group. Dietary supplementation of DKSM (DKSM-Low and DKSM-High groups), PSRE (PSRE-Low and PSRE-High groups), and simvastatin (Statin group) exerted superior antihypercholesterolemic properties in the rats, with significant suppressions of elevated total and LDL cholesterol levels. Besides, supplementation of DKSM and PSRE significantly enhanced the high-density lipoprotein (HDL) cholesterol level of hypercholesterolemic rats. Furthermore, supplementations of DKSM, PSRE, and simvastatin had successfully improved the hepatosteatosis of hypercholesterolemic rats by the significant lowering of hepatosomatic indexes and hepatic lipid contents.

### 2.4. Serum Total Antioxidant Capacity

Serum total antioxidant capacity of experimental rats was assessed using a modified Trolox equivalent antioxidant capacity (TEAC) assay described by Katalinic et al. [25] and Chan et al. [18]. ABTS^•+^ stock solution was prepared by reacting 7 mM of ABTS with 2.45 mM of potassium persulfate. After 18 h of incubation in the dark at room temperature, the stock solution was diluted with phosphate buffer saline to the absorbance of 0.70 ± 0.02 at 734 nm (PharmaSpec UV-1700, Shimadzu, Kyoto, Japan). Subsequently, 50 *μ*L of diluted serum was reacted with 950 *μ*L of adjusted ABTS^•+^ solution for 10 min, and the absorbance was measured at 734 nm (PharmaSpec UV-1700, Shimadzu, Kyoto, Japan). Trolox was used as standard, and the serum total antioxidant capacity of experimental rats was expressed as mg Trolox equivalent antioxidant capacity (TEAC)/mL serum.

### 2.5. Hepatic mRNA Levels of Antioxidant Genes

The primers for the gene expression study were designed by referring to the *Rattus norvegicus* gene sequences from the National Center for Biotechnology Information website (http://www.ncbi.nlm.nih.gov/nucleotide/) and tagged with an 18-nucleotide universal forward and 19-nucleotide universal reverse sequence, respectively. Primers were supplied by Integrated DNA Technologies (Singapore) and reconstituted in RNAse-free water. The primer sequences of 5 antioxidant genes, 3 housekeeping genes, and an internal control (Kanr) for the rat hepatic multiplex panel are shown in Table 2.

Rat hepatic RNA was extracted using an RNA isolation kit according to the manufacturer's instructions, while the processes of reverse transcription (RT) and polymerase chain reaction (PCR) were conducted according to the GenomeLab™ GeXP Start Kit protocol. Multiplex universal reverse primers and 50 ng extracted RNA were used for RT in an XP Thermal Cycler (BIOER Technology, Hangzhou, Zhejiang, China) under the following conditions: 48°C for 1 min, 37°C for 5 min, 42°C for 60 min, 95°C for 5 min, and then held at 4°C. Subsequently, the cDNA product (9.3 *μ*L) was mixed with 2 *μ*L of 200 nM forward universal primers, 4 *μ*L 25 mM MgCl_2_, 0.7 *μ*L of Thermo Start Taq DNA polymerase, and 4 *μ*L of 5x PCR Master Mix buffer and subjected to PCR in an XP Thermal Cycler (BIOER Technology, Hangzhou, Zhejiang, China) under the following conditions: initial denaturation at 95°C for 10 min, followed by two-step cycles of 94°C for 30 s and 55°C for 30 s, ending in a single-extension cycle of 68°C for 1 min.

The PCR products obtained from previous steps were analyzed using GeXP GenomeLab Genetic Analysis System (Beckman Coulter Inc., Brea, CA, USA). In brief, 1 *μ*L of PCR products was mixed with 38.5 *μ*L sample loading solution and 0.5 *μ*L DNA size standard 400 (provided in the GenomeLab GeXP Start Kit) on a 96-well sample plate before loading on the machine. Results were analysed with the Fragment Analysis Module of the GeXP system software and normalized on the Express Profiler software.

### 2.6. Circulating Oxidized Low-Density Lipoprotein and Proinflammatory Biomarkers

Fasting sera of rats were subjected to immunoassays (ELISA kits) for determination of circulating oxidized LDL (oxLDL) and proinflammatory biomarkers according to the manufacturer's instructions. The levels of circulating oxLDL and C-reactive protein (CRP) were expressed in ng/mL and *μ*g/mL serum, respectively, while levels of circulating tumour necrosis factor-alpha (TNF-*α*) and interleukin 6 (IL-6) were determined as pg/mL serum.

### 2.7. Statistical Analysis

All results are reported as mean ± standard deviation (*n* = 6). One-way analysis of variance (ANOVA), accompanied with Tukey's multiple comparison test (GraphPad Prism 6.01, GraphPad Software Inc., La Jolla, CA, USA), was conducted to identify significant differences between samples (*p* < 0.05).

## 3. Results and Discussion

### 3.1. Serum Total Antioxidant Capacity

Oxidative stress is closely associated with the pathogenesis of atherosclerosis [6]. Thus, serum/plasma total antioxidant capacity (TAC) may represent a useful tool in assessing the global oxidative stress and antioxidant defense levels in experimental animals and human subjects [26–30]. Trolox equivalent antioxidant capacity (TEAC) assay is one of the most common assays employed in the assessment of serum TAC based on the spectrophotometric measurement of ABTS^•**+**^ cation reduction (decay of green-blue chromophore absorbance) by serum antioxidative components, in comparison to the control antioxidant, Trolox (hydrophilic analogue of vitamin E) [31].

**Figure 1:**
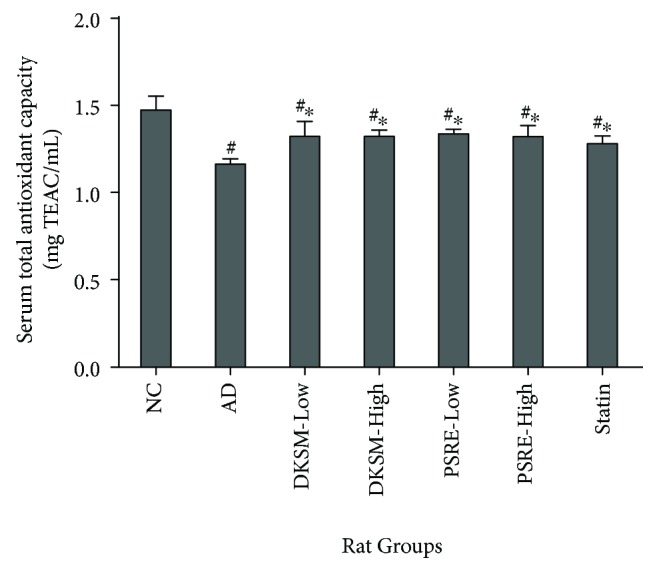
Serum total antioxidant capacity after 10 weeks of dietary intervention. Data represent the mean of each group, accompanied with standard deviation. Symbols “∗” and “#” respectively represent significant difference between samples in comparison to the AD and NC groups (*p* < 0.05).

Figure 1 depicts serum TAC of experimental rats after 10 weeks of dietary intervention. Atherogenic diet feeding significantly lowered serum TAC of hypercholesterolemic rats in the AD group (*p* < 0.05). This finding is in agreement with several *in vivo* studies involving diet-induced hypercholesterolemic/hyperlipidemic rats [10, 32, 33]. The depletion of serum TAC was probably due to the override of *in vivo* antioxidant defense by excessive generation of oxidants/ROS under hypercholesterolemic condition. In contrast, simvastatin treatment significantly improved serum TAC of hypercholesterolemic rats (*p* < 0.05), affirming the pleiotropic antioxidant properties of simvastatin [34].

As compared to the AD group, supplementation of DKSM and PSRE effectively counteracted the decrease in serum TAC induced by hypercholesterolemia (*p* < 0.05). The improvement in serum TAC in hypercholesterolemic rats was probably due to high antioxidant properties of DKSM and PSRE, which is supported by our previous studies using *in vitro* assays based on different mechanisms [18, 19]. Consumption of an antioxidant-rich diet (e.g., fruits and vegetable which are rich in polyphenols) is strongly correlated with the improvement of antioxidant status and the attenuation of atherogenic risks in human subjects [28, 35–37].

In the present study, phenolics and saponins may have substantially contributed to the *in vivo* antioxidant properties of DKSM and PSRE since serum TAC between the DKSM groups and their corresponding PSRE groups (which contained the equivalent levels of DKSM-phenolics and saponins with the DKSM groups) were insignificantly different (*p* > 0.05). In agreement to our previous study [18], phenolics and saponins had significantly contributed to the antioxidant properties of PSRE and DKSM. This postulation is further supported by a number of studies reporting on the *in vivo* antioxidant properties of major phenolic compounds detected in DKSM and PSRE, that is, *p*-coumaric acid, caffeic acid, (+)-catechin, and gallic acid. For instance, oral administration of *p*-coumaric acid and gallic acid at the dosage of 100 mg/kg body weight for 2 weeks was found to greatly improve the cardiac and hepatic total antioxidant capacities of healthy rats [38, 39], while dietary supplementation with caffeic and coumaric acids (0.2% of total diet) for 6 weeks effectively enhanced the *in vivo* antioxidant capacity of hypercholesterolemic rats [40]. On the other hand, plasma and urine TAC of Wistar rats was significantly increased following 10 days of intraperitoneal administration of catechin mixture (23 mg/kg body weight) [41]. Besides phenolic compounds, saponins have also been proposed as a group of dietary phytochemicals with distinctive *in vivo* antioxidant properties [42, 43]. For example, supplementation with total saponins extracted from three medicinal species of *Dioscorea* and dry root tuber of *Trichosanthis kirilowii* were found to effectively improve the *in vivo* antioxidant capacity of myocardial ischemic rats and carbon tetrachloride-intoxicated mice, respectively [44, 45].

### 3.2. Expression of Hepatic Antioxidant Genes

Endogenous antioxidant defense plays a critical role in restoring the cellular redox imbalance caused by oxidative insults, whilst consumption of high antioxidative phytochemicals (particularly phenolic compounds) has been implicated in the enhancement of endogenous antioxidant defense via modulation of multiple redox mechanisms [46, 47].  Table 3 shows the mRNA levels of hepatic antioxidant genes (nuclear factor erythroid 2-related factor 2 (Nrf2 or Nfe2l2), cytosolic superoxide dismutase (Sod1), mitochondrial superoxide dismutase (Sod2), glutathione-disulfide reductase (Gsr), and glutathione peroxidase 1 (Gpx1)) in the experimental rats, as influenced by different dietary interventions.

After 10 weeks of atherogenic diet feeding, hepatic antioxidant gene expression (Nrf2, Sod1, Sod2, Gsr, and Gpx1) in the AD group was adversely altered (*p* < 0.05) in comparison to the NC group, suggesting the manifestation of hypercholesterolemia-induced oxidative stress in these rats. On the contrary, hepatic antioxidant gene expressions in the Statin group were significantly enhanced (*p* < 0.05). In consonance with previous studies, diet-induced hypercholesterolemia has been associated with the exacerbation of oxidative stress and compromised endogenous antioxidant defense in the experimental animals, while statin treatment is the effective pharmaceutical approach in reversing these deleterious impacts [10, 13, 32, 48, 49].

In the present study, supplementation with DKSM and PSRE substantially improved the endogenous antioxidant defense of hypercholesterolemic rats via transcriptional modulation of hepatic antioxidant genes. In comparison to the AD group, hepatic Nrf2 gene expression of all DKSM- and PSRE-supplemented rats was significantly elevated by 1.5- to 1.8-folds (*p* < 0.05). DKSM supplementation resulted in the upregulation of hepatic Sod1, Sod2, and Gsr expressions, especially in the DKSM-High group (*p* < 0.05). However, supplementation with DKSM did not improve the hepatic Gpx1 gene expression of hypercholesterolemic rats (*p* > 0.05). Similarly, PSRE supplementation upregulated the hepatic gene expressions of Sod1, Sod2, Gsr, and Gpx1 of hypercholesterolemic rats by 2- to 3-folds (*p* < 0.05). Except for Nrf2, both PSRE groups exhibited superior upregulatory effects in the expressions of all studied hepatic antioxidant genes than their corresponding DKSM groups, which contained an equivalent level of total DKSM-phenolics and saponins (*p* < 0.05). This is probably due to the enhancement in the release of bioactives (phenolics and saponins) from the DKSM matrix during the heated reflux extraction process, which may then result in the better bioavailability and bioefficiency of PSRE. Appropriate increase in the extraction temperature will disrupt the integrity of the cell wall, thus facilitating the release of bound bioactives from the matrix and enhancing the solubility and diffusion coefficient of bioactives into the extraction solvent, leading to the optimal recovery of bioactives in the extract [50, 51]. For instance, heated reflux extraction (80°C) of *Pterodon emarginatus* vogel seeds with 70% aqueous ethanol provided the highest phenolic recovery as compared to the nonthermal extraction processes [52], while a higher recovery of chickpeasaponin B1 was observed when the aqueous-ethanolic extraction processes were carried out under heated reflux condition (90°C) in comparison to the nonthermal ultrasonic extraction [53].

In the present study, results from hepatic antioxidant gene expression analysis aggregately suggest that supplementation with DKSM and PSRE could have activated the hepatic Nrf2-ARE pathway in the experimental rats and consequently improved their endogenous antioxidant defense against hypercholesterolemia-induced oxidative stress. Besides, supplementation with PSRE at an equivalent level of DKSM-phenolics and saponins produced similar or superior modulatory effects on the hepatic antioxidant gene expressions than on their corresponding DKSM counterparts. This finding signifies the contributory roles of phenolics and saponins as the dietary Nrf2-ARE-activating factors in DKSM and PSRE. In recent years, activation of Nrf2-ARE has been proposed as the targeted therapeutic pathway for a wide array of degenerative and immunological diseases, particularly CVDs, whilst a number of dietary phytochemicals especially polyphenols, isothiocyanates, organosulfur compounds, saponins, and curcumin are prominent natural activators of this pathway [46, 54–56]. Nrf2 is a critical transcription factor that regulates the antioxidant responses against oxidative insults. Once it is activated, Nrf2 binds to the antioxidant response element (ARE) in the nucleus to upregulate a vast array of antioxidative and electrophile detoxification genes, such as Sod, Gsr, and Gpx [57]. Antioxidant enzymes are the core pillars of endogenous antioxidant defense that cohesively shield our body from oxidative damage and its related pathogenesis [58]. For instance, Sod is one of the most effective primary antioxidant enzymes that catalyses the conversion of superoxide anions to hydrogen peroxide, while Gpx renders hydrogen peroxide and other organic hydroperoxides (e.g., lipid peroxide) into inert end products. On the other hand, Gsr serves as an important secondary antioxidant enzyme that maintains the proper function of primary antioxidant enzymes (e.g., Gpx) by catalysing the reduction process of glutathione disulfide (GSSG) to glutathione (GSH) with NADPH as the reducing cofactor.

Interestingly, simvastatin treatment and PSRE supplementation did not only neutralize the deleterious effects of hypercholesterolemia on the transcriptions of hepatic antioxidant genes but their hepatic expressions of antioxidant genes were upregulated to a higher degree than those of the NC group (*p* < 0.05). In the present study, PSRE supplementation and simvastatin treatment significantly improved the severity of hypercholesterolemia in the experimental rats and thus produced a milder oxidative stress condition as compared to the AD group. Mild/moderate oxidative stress, simvastatin, and polyphenols have been previously reported as the activators of the Nrf2-ARE pathway by inducing the dissociation of Nrf2 from the Kelch-like ECH-associated protein-1 (Keap1) and consequently upregulating the expressions of its downstream antioxidant genes [55, 59, 60]. Although the hepatic Nrf2 expression between the NC and PSRE groups were indifferent (*p* > 0.05), the higher expressions of other hepatic antioxidant genes in the PSRE and Statin groups could be possibly explained by the enhancement of Nrf2-ARE activation through the combinatorial effects of improved oxidative stress condition as well as the inductions by simvastatin or PSRE bioactives. In agreement, the similar findings have been previously observed in the diet-induced hyperlipidemic rats, supplemented with phenolic-rich extract from *Clinacanthus nutans* and simvastatin [10].

### 3.3. Circulating Oxidized Low-Density Lipoprotein and Proinflammatory Biomarkers

Chronic hypercholesterolemia triggers excessive ROS generation, compromises endogenous antioxidant defense, and consequently results in the formation of oxidatively modified LDL/oxidized LDL (oxLDL) [4]. Circulating oxLDL level is one of the most important oxidative stress-related biomarkers, which is strongly correlated to the prevalence of atherosclerotic CVD [61–63]. Circulating oxLDL and proinflammatory biomarker levels of experimental rats after 10 weeks of dietary intervention are depicted in Table 4. In comparison to the NC group, atherogenic diet feeding significantly elevated the circulating oxLDL level of the AD group (*p* < 0.05), indicating the successful induction and advanced manifestation of hypercholesterolemia-induced oxidative stress in these rats. In contrast, dietary supplementation with DKSM and PSRE effectively lowered the circulating oxLDL level of hypercholesterolemic rats by 34% to 57%, in a dose-dependent manner of DKSM-High ≥ PSRE-High ≥ Statin ≥ DKSM-Low ≥ PSRE-Low > AD (*p* < 0.05). Remarkably, supplementation with higher concentration of DKSM (DKSM-High group) exhibited superior LDL oxidation inhibitory activity than simvastatin treatment (*p* < 0.05). Since there was no significant difference in the circulating oxLDL levels between the DKSM groups and their corresponding PSRE groups (*p* > 0.05), it is suggested that phenolics and saponins could have contributed to the antioxidant and LDL oxidation inhibitory properties of DKSM and PSRE. This deduction is further supported by our previous studies, of which the phenolic-saponin-rich fraction obtained via partial purification of DKSM ethanolic extract exhibited superior antioxidant properties than its bioactive-deficient counterpart [18]. Furthermore, some of the major phenolics in DKSM and PSRE, that is, *p*-coumaric acid, caffeic acid, and (+)-catechin, have been previously reported as promising natural inhibitors against LDL oxidation via *in vitro* and *in vivo* models [64–67].

Due to the critical roles of circulating TNF-*α*, IL-6, and CRP in the pathogenesis of atherosclerosis, these proinflammatory biomarkers are frequently used as promising panel for the assessment of cardiovascular risks [68, 69]. In the present study, hypercholesterolemia had evidently induced systemic inflammation in the experimental rats fed on an atherogenic diet. In comparison to the NC group, significant elevations of circulating TNF-*α*, IL-6, and CRP levels by 4-, 1.4-, and 1.3-folds, respectively, were observed in the hypercholesterolemic rats from the AD group (*p* < 0.05). After 10 weeks of DKSM and PSRE supplementation, circulating TNF-*α* levels of hypercholesterolemic rats were markedly reduced by 39 to 60% in a dose-dependent order, that is, PSRE-High ≥ DKSM-High ≥ DKSM-Low ≥ PSRE-Low ≥ Statin > AD (*p* < 0.05). A significantly lower circulating IL-6 level in the experimental rats was observed in the DKSM-High (−33.4%), PSRE-Low (−23.5%), and PSRE-High (−18.5%) groups in comparison to the AD group (*p* < 0.05), while simvastatin treatment (Statin group) and DKSM supplementation at a lower level (DKSM-Low group) produced insignificant lowering effects on the circulating IL-6 level (*p* > 0.05). Similar to the trend of the TNF-*α* level, supplementation with DKSM and PSRE effectively repressed the circulating CRP level of hypercholesterolemic rats in a dose-dependent manner, that is, DKSM-High ≥ Statin ≥ PSRE-High ≥ PSRE-Low ≥ DKSM-Low > AD (*p* < 0.05). Furthermore, there were no significant differences in the levels of proinflammatory biomarkers between DKSM groups and their corresponding PSRE groups (*p* > 0.05), suggesting that phenolics and saponins might be the key bioactives that have contributed to the anti-inflammatory properties of DKSM and PSRE.

Elevated level of oxLDL is correlated with the upregulation of proinflammatory mediators (e.g., TNF-*α*, IL-6, and CRP) in human subjects [62]. In the present study, the level of circulating oxLDL was strongly correlated with the level of CRP (*r* = 0.9390) and moderately correlated with the TNF-*α* (*r* = 0.5682) and IL-6 (*r* = 0.5892) levels, thus affirming the etiological role of hypercholesterolemia-induced oxidative stress in eliciting systemic inflammation and higher atherogenic risk in the rats. Moreover, these correlations also suggest that the lower systemic inflammation observed in DKSM- and PSRE-supplemented rats may in part be due to the inhibition of LDL oxidation by these dietary interventions. Supplementation with DKSM and PSRE modulated the upregulation of hepatic antioxidant gene expressions as well as the enhancement of circulating nonenzymatic low molecular weight antioxidant levels (as evidenced by improvement in serum TAC) in the hypercholesterolemic rats. Thus, it is postulated that these antioxidative effects might have advantageously controlled hypercholesterolemia-induced ROS overproduction in the rats and therefore reduced the severity of LDL oxidative damage and systemic inflammation. Although PSRE supplementation showed superior upregulatory effects than its DKSM counterpart in the hepatic expressions of antioxidant genes, similar effects were not observed in TAC, oxLDL, and proinflammatory biomarker assays. This is possibly due to the relatively high levels of DKSM and PSRE used in the present study, hence resulting in the optimal *in vivo* antioxidant and anti-inflammatory effects (i.e., plateau portion of the dose-response curve) observed under the tested physiological condition. On the other side, DKSM and PSRE supplementation might have exhibited an all-or-none effect notwithstanding the transcriptional changes in the *in vivo* model. This is probably due to the posttranscriptional modifications that produced therapeutic effects to the same degree irrespective of the degree of transcriptional changes induced. Investigations on dietary effects of DKSM and PSRE on endogenous antioxidant defense in hypercholesterolemic animal models at posttranscriptional and translational levels are suggested for further studies.

## 4. Conclusion

DKSM and its derived PSRE supplementation improved *in vivo* antioxidant defense of hypercholesterolemic rats possibly via transcriptional activation of hepatic Nrf2-ARE pathway and improvement of serum TAC. The enhancement in endogenous antioxidant defense therefore meritoriously inhibited the oxidation of LDL and systematic inflammatory response in the hypercholesterolemic rats. Phenolics and saponins are suggested as the key antioxidant and anti-inflammatory bioactives in DKSM and PSRE. Finally, DKSM and PSRE could be potentially used as cardioprotective functional food ingredients in counteracting hypercholesterolemia-associated oxidative stress and systemic inflammation.

## Figures and Tables

**Table 1:** Composition (g/100 g diet) and energy distribution of diets.

	Rat Groups					
	NC	AD/Statin	DKSM-Low	DKSM-High	PSRE-Low	PSRE-High
*Ingredient*						
Ground standard rat chow	100.0	60.0	45.0	30.0	57.7	55.4
DKSM			15.0	30.0		
PSRE					2.3^∗^	4.6^#^
Palm oil		20.0	20.0	20.0	20.0	20.0
Full cream milk powder		15.0	15.0	15.0	15.0	15.0
Egg yolk		1.5	1.5	1.5	1.5	1.5
Cholesterol		2.0	2.0	2.0	2.0	2.0
Cholic acid		0.4	0.4	0.4	0.4	0.4
Starch		1.1	1.1	1.1	1.1	1.1
Total	100.0	100.0	100.0	100.0	100.0	100.0
*Energy distribution*						
Protein (% kcal)	22.2	13.2	14.1	15.0	12.9	12.5
Carbohydrate (% kcal)	65.8	35.7	35.6	35.5	36.0	36.4
Fat (% kcal)	12.0	51.1	50.3	49.5	51.1	51.1
Total caloric value (kcal/100 g diet)	360.8	479.3	476.1	473.0	478.2	477.0

^∗^Based on 15.36% (*w*/*w*) extraction yield, which corresponds to the equivalent level of total DKSM-phenolics and saponins in the “DKSM-Low” group; ^#^based on 15.36% (*w*/*w*) extraction yield, which corresponds to the equivalent level of total DKSM-phenolics and saponins in the “DKSM-High” group.

**Table 2:** Gene name, accession number, and primer sequences used in GeXP multiplex analysis.

Gene name	Accession number	Primer sequence (with universal tag)	
		Forward	Reverse
Nrf2/Nfe2l2	NM_031789.2	AGGTGACACTATAGAATATCAGTTACAACTGGATGAAG	GTACGACTCACTATAGGGAGACTCATGGTCATCTACAAAT
Sod1	NM_017050	AGGTGACACTATAGAATAATATGGGGACAATACACAA	GTACGACTCACTATAGGGATCCAACATGCCTCTCT
Sod2	NM_017051	AGGTGACACTATAGAATACAGGTTGCTCTTCAGC	GTACGACTCACTATAGGGAAACTCTCCTTTGGGTTCT
Gsr	NM_053906.2	AGGTGACACTATAGAATAAATAAACTGGGGATTCAGAC	GTACGACTCACTATAGGGAAGTAGATTTTCACATTGTCTTTG
Gpx1	NM_030826	AGGTGACACTATAGAATATTGAGAAGTTCCTGGTAGGT	GTACGACTCACTATAGGGATTTTCTGGAAATCAGGTGT
B2m^a^	NM_012512	AGGTGACACTATAGAATAATGCTTGCAGAGTTAAACA	GTACGACTCACTATAGGGATGCATAAAATATTTAAGGTAAGA
Kan(r)^c^		GGTGACACTATAGAATAATCATCAGCATTGCATTCGATTCCTGTTTG	GTACGACTCACTATAGGGAATTCCGACTCGTCCAACATC
Hprt1^a,b^	NM_012583	AGGTGACACTATAGAATATCCTCATGGACTGATTATG	GTACGACTCACTATAGGGACTGGTCATTACAGTAGCTCTT
Rpl13a^a^	NM_173340	AGGTGACACTATAGAATAATGGGATCCCTCCAC	GTACGACTCACTATAGGGAATTTTCTTCTCCACATTCTT

^a^Housekeeping genes; ^b^normalization gene; ^c^internal control supplied by Beckman Coulter Inc. (Brea, CA, USA).

**Table 3:** Hepatic antioxidant gene expressions after 10 weeks of dietary intervention.

Rat groups	Hepatic antioxidant genes (relative expression)				
	Nrf2	Sod1	Sod2	Gsr	Gpx1
NC	1.00 ± 0.09	1.00 ± 0.11	1.00 ± 0.20	1.00 ± 0.20	1.00 ± 0.11
AD	0.68 ± 0.19^#^	0.56 ± 0.10^#^	0.67 ± 0.12^#^	0.57 ± 0.11^#^	0.61 ± 0.12^#^
DKSM-Low	1.21 ± 0.08^∗^	0.76 ± 0.12^∗^^#^	0.88 ± 0.12	0.82 ± 0.08^∗^	0.50 ± 0.04^#^
DKSM-High	1.23 ± 0.18^∗^^#^	0.76 ± 0.09^∗^^#^	1.02 ± 0.17^∗^	0.83 ± 0.12^∗^	0.66 ± 0.09^#^
PSRE-Low	1.02 ± 0.16^∗^	1.57 ± 0.09^∗^^#^	2.09 ± 0.17^∗^^#^	1.28 ± 0.04^∗^^#^	1.71 ± 0.32^∗^^#^
PSRE-High	1.16 ± 0.17^∗^	1.53 ± 0.11^∗^^#^	2.18 ± 0.40^∗^^#^	1.37 ± 0.17^∗^^#^	1.64 ± 0.29^∗^^#^
Statin	1.69 ± 0.04^∗^^#^	1.75 ± 0.03^∗^^#^	1.74 ± 0.12^∗^^#^	1.42 ± 0.23^∗^^#^	2.04 ± 0.28^∗^^#^

Symbol “∗” within the same column indicates significant difference in comparison to the AD group (*p* < 0.05); symbol “#” within the same column indicates significant difference in comparison to the NC group (*p* < 0.05); abbreviations: Nrf2: nuclear factor erythroid 2-related factor 2; Sod1: cytosolic superoxide dismutase; Sod2: mitochondrial superoxide dismutase; Gsr: glutathione-disulfide reductase; Gpx1: glutathione peroxidase 1.

**Table 4:** Circulating oxidized low-density lipoprotein and proinflammatory biomarkers after 10 weeks of dietary intervention.

Rat groups	oxLDL (ng/mL)	TNF-*α* (pg/mL)	IL-6 (pg/mL)	CRP (*μ*g/mL)
NC	26.90 ± 2.29	20.75 ± 3.86	3.08 ± 0.18	798.42 ± 55.95
AD	35.90 ± 3.71^#^	83.00 ± 19.08^#^	4.22 ± 0.20^#^	1018.56 ± 155.09^#^
DKSM-Low	21.49 ± 0.83^∗^^#^	46.75 ± 7.72^∗^^#^	3.66 ± 0.25	749.31 ± 42.60^∗^
DKSM-High	15.35 ± 2.85^∗^^#^	45.25 ± 9.78^∗^^#^	2.81 ± 0.40^∗^	624.41 ± 42.07^∗^^#^
PSRE-Low	23.79 ± 1.72^∗^	50.50 ± 8.39^∗^^#^	3.23 ± 0.30^∗^	727.67 ± 67.66^∗^
PSRE-High	16.81 ± 1.34^∗^^#^	33.00 ± 2.00^∗^	3.44 ± 0.88^∗^	725.22 ± 54.81^∗^
Statin	20.85 ± 5.64^∗^^#^	52.50 ± 6.56^∗^^#^	3.92 ± 0.68^#^	683.39 ± 77.96^∗^

Symbol “∗” within the same column indicates significant difference in comparison to the AD group (*p* < 0.05); symbol “#” within the same column indicates significant difference in comparison to the NC group (*p* < 0.05); abbreviations: oxLDL: oxidized low-density lipoprotein; TNF-*α*: tumour necrosis factor-alpha; IL-6: interleukin 6; CRP: C-reactive protein.
